# Integrated transcriptome and hormone profiling highlight the role of multiple phytohormone pathways in wheat resistance against fusarium head blight

**DOI:** 10.1371/journal.pone.0207036

**Published:** 2018-11-07

**Authors:** Lipu Wang, Qiang Li, Ziying Liu, Anu Surendra, Youlian Pan, Yifeng Li, L. Irina Zaharia, Thérèse Ouellet, Pierre R. Fobert

**Affiliations:** 1 National Research Council Canada, Saskatoon, SK, Canada; 2 Department of Plant Sciences, University of Saskatchewan, Saskatoon, SK, Canada; 3 National Research Council Canada, Ottawa, ON, Canada; 4 Ottawa Research and Development Centre, Agriculture and Agri-Food Canada, Ottawa, ON, Canada; Institute of Genetics and Developmental Biology Chinese Academy of Sciences, CHINA

## Abstract

Fusarium head blight (FHB or scab) caused by *Fusarium* spp. is a destructive disease of wheat. Since the most effective sources of FHB resistance are typically associated with unfavorable agronomic traits, breeding commercial cultivars that combine desired agronomic traits and a high level of FHB resistance remains a considerable challenge. A better understanding of the molecular mechanisms governing FHB resistance will help to design more efficient and precise breeding strategies. Here, multiple molecular tools and assays were deployed to compare the resistant variety Sumai3 with three regionally adapted Canadian cultivars. Macroscopic and microscopic disease evaluation established the relative level of Type II FHB resistance of the four varieties and revealed that the *F*. *graminearum* infection process displayed substantial temporal differences among organs. The rachis was found to play a critical role in preventing *F*. *graminearum* spread within spikes. Large-scale, organ-specific RNA-seq at different times after *F*. *graminearum* infection demonstrated that diverse defense mechanisms were expressed faster and more intensely in the spikelet of resistant varieties. The roles of plant hormones during the interaction of wheat with *F*. *graminearum* was inferred based on the transcriptomic data obtained and the quantification of the major plant hormones. Salicylic acid and jasmonic acid were found to play predominantly positive roles in FHB resistance, whereas auxin and ABA were associated with susceptibility, and ethylene appeared to play a dual role during the interaction with *F graminearum*.

## Introduction

Fusarium head blight (FHB), primarily caused by *Fusarium graminearum* Schwabe (teleomorph *Gibberella zeae* (Schweinitz) Petch), is one of the most destructive diseases of wheat (*Triticum aestivum* L.) [1]. The fungus invades wheat spike tissues and causes disease through a series of complex processes and mechanisms. In general, airborne *F*. *graminearum* spores attach and germinate onto flowering spikelets. Subcuticular and intercellular growth of *F*. *graminearum* has been observed during the first 2 days post infection (dpi), suggesting a biotrophic phase in advance of necrotization of the plant tissue by intracellular hyphae [2–4]. Necrotrophic growth and nutrition is facilitated by the secretion of proteases and mycotoxins, including deoxynivalenol (DON) [5]. At 3 or 4 dpi, extensive, unbranched inter- and intracellular hyphae are present throughout the ovary and floral brackets, which subsequently display dark-brown, water-soaked symptoms [2, 6]. After 5 dpi, *F*. *graminearum* hyphae pass the rachilla of the inoculated spikelet and enter the cortex of the rachis node [7]. Several days after entry into the rachis, *F*. *graminearum* hyphae invade uninoculated spikelets, resulting in severe dark-brown, water-soaked disease symptoms that eventually cause premature bleaching of the wheat spike [8].

Host resistance is broadly considered as the most economical, ecofriendly and efficient approach to control FHB [1]. Three or more major types of FHB resistance are recognized and used in wheat breeding programs: resistance to initial infection (type I); resistance to spread within a spike (type II); and resistance to mycotoxin accumulation in grain (type III). Although substantial progress has been made in understanding the molecular mechanisms governing FHB resistance, much remains elusive. Analysis has been complicated by the complex genetic base of FHB resistance, governed by a large number of quantitative trait loci (QTL), and the elaborate structure of the wheat spike that possesses multiple organs and undergoes various developmental processes following infection by *F*. *graminearum* [9]. These developmental processes are largely regulated through networks coordinated by plant hormones [10]. Alteration of hormonal balances may result in poor spikelet development [11], reduction in grain weight [12] and quality in wheat [13]. Several hormone pathways also regulate immune responses to microbial pathogens [14]. To survive, plant cells reallocate limited resources and energy from growth processes to deal with pathogen attack [15], phenomena called growth-defense tradeoffs, which are largely regulated by fine-tuning of hormones effects to achieve optimum fitness. Intriguingly, successful pathogens have evolved mechanisms to manipulate or subvert plant hormone signaling pathways to facilitate pathogen infection and disease development [16].

Most existing FHB resistance is associated with other unfavorable agronomic traits [1]. In-depth investigation of molecular interactions between *F*. *graminearum* and wheat will help us to better understand this complex trait and to design more efficient and precise breeding strategies. In this study, the molecular mechanisms of FHB resistance were investigated by comparing the resistant variety Sumai3 with three regionally adapted Canadian cultivars. The extent of FHB resistance in four varieties was determined macroscopically and microscopically through a comprehensive disease evaluation strategy. Large-scale, organ-specific RNA-seq and hormone profiling experiments were performed on the four varieties after *F*. *graminearum* infection. Compared with “bulk material”, organ-specific analyses prevent averaged information and diluted signals, and provide more accurate and precise information [17]. This is particularly important for *F*. *graminearum* infected wheat spikes, which are composed of multiple organs. From this analysis, the role of several plant hormones is proposed during interaction with *F*. *graminearum*, including salicylic acid (SA), jasmonic acid (JA), ethylene (ET), auxin and abscisic acid (ABA).

## Material and methods

### Plant material and growth conditions

All experiments were conducted in environment-controlled growth chambers. Seeds were sown in peat pots (diameter, 12.7 cm) and maintained in a growth chamber at 21°C/19°C: day/night cycle, with a 16 h photoperiod. Plants were fertilized weekly with 20-20-20 (N-P-K).

Four cultivars are used in this study. Sumai3 is among the most resistant cultivars, but displays unacceptably low yield potential and low end-use quality [1]. The Canadian Western Red Spring (CWRS) cultivars, Stettler, developed in 2006 and Muchmore, released in 2009, have been widely cultivated in Western Canada due to their high yield and quality [18, 19]. However, both are moderately susceptible to FHB [18, 19]. The third Canadian elite line tested, FL62R1 from Eastern Canada, displayed a near Sumai3 level of resistance in the field [20], but moderate Type II resistance in the greenhouse [21, 22].

### Fungal growth and inoculation

A Western Canadian isolate of *F*. *graminearum*, 3-ADON chemotype (M9-4-6), originally obtained from Dr. J. Gilbert at the Agriculture and Agri-Food Canada Cereal Research Centre in Winnipeg, MB, was used throughout. Fungal growth and inoculation assays were as described previously [21]. Briefly, fresh cultures were routinely propagated on Potato Dextrose Agar (PDA, Difco) medium incubated at room temperature. Conidia were produced in CarboxyMethyl Cellulose (CMC) medium at 28°C, 180 rpm for 2 days. Conidial suspensions were harvested in sterile water, filtered through cheesecloth, and concentrations determined with a hemocytometer by microscopy.

At mid-anthesis, single floret inoculation was carried out by pipetting 10 μl of the macroconidial suspension (5x10^4^ spores ml^-1^) between the palea and lemma. Inoculated plants were covered with a plastic bag for 2 days. For disease severity tests and microscopic observation, a pair of alternate spikelets in the middle of the head was inoculated. The number of infected rachis internodes and spikelets from the inoculated site was recorded over a three-week period. Two spikes per plant and 20 plants per variety were examined. The experiment was repeated three times with similar results. Data from each time point were analyzed using a one-way Analysis of Variance (ANOVA), General Linear Model (SAS Institute Inc., http://www.sas.com), and differences between varieties determined by post-hoc testing (Tukey’s Honest Significant Difference test).

For RNA-seq and hormone analysis experiments, 10 spikelets midway along the spike were point inoculated with 10 μl of the macroconidial suspension as described above or with 10 μl ddH_2_O as a mock treatment. Three spikes from each variety were pooled as one biological replicate and three biological replicates were harvested per time point. Each replicate was ground and divided into two parts for separate processing and analysis by RNA-seq and hormone profiling, respectively.

### Microscopic observation

The infected spikes were individually excised and fixed in a solution of 60% methanol, 30% chloroform and 10% acetic acid. After rehydration, the material was stained with Wheat Germ Agglutinin (WGA), Alexa Fluor 488 conjugate (Invitrogen, USA) and examined by fluorescence stereomicroscopy (SteREO Lumar.V12, Zeiss). Photos were taken with a Zeiss AxioCam HR colored camera.

### RNA sequencing and data processing

Total RNA from each sample was isolated using the Plant RNeasy Mini Kit (Qiagen, Mississauga, ON, Canada), according to the manufacturer’s instructions. All samples were treated with DNase I on column using the Qiagen RNase-Free DNase Set. The yield and RNA purity were determined spectrophotometrically with a Nanodrop 1100 (Thermo scientific, USA), and the quality of RNA was monitored by determining the RNA Integrity Number (RIN) with an Agilent 2100 bioanalyzer and RNA LabChip (Agilent Technologies, Santa Clara, CA, USA). Samples with a RIN value > = 7 were used to prepare cDNA libraries by using the TruSeq RNA Sample Preparation Kit v2 (Illumina). Paired-end sequencing was conducted on the Illumina HiSeq2500, generating 101-nucleotide reads, at the National Research Council, ACRD-Saskatoon, Canada.

Sequencing adapters were removed and low-quality reads were trimmed following the methods described in [23]. The filtered reads were mapped to the wheat genome survey v2.2 sequence obtained from URGI (http://wheat-urgi.versailles.inra.fr/) (International Wheat Genome Sequencing Consortium, 2014) and read counts per gene were estimated using STAR (v2.4.2a) [24].

### Differential gene expression analysis

Normalization and differential expression analysis was performed with DESeq2 [25]. After calling for differentially expressed genes (DEGs), the normalized data along with log 2 fold changes (log2FC), p-values, and adjusted p-values were saved for downstream analysis. The data were reduced in size to a set of DEGs. Genes were determined to be significantly differentially expressed if they had a log2 (fold change) > 1 or < - 1 and a P <0.01.

### Principal component analysis

Principal component analysis (PCA) was performed using the *prcomp* function from the R base package. Malhalanobis distances and associated p-values were calculated using the *pca-utils* software tools [26].

### Gene ontology annotations and enrichment analysis

Gene Ontology (GO) annotations for mRNA transcript sequences were derived from databases containing orthology sequences of *Arabidopsis thaliana*, *Brachypodium distachyon*, *Oryza sativa* Japonica, and *Zea mays* (EnsemblPlants version 25). GO enrichment analysis was performed by using GOAL software ([27]). The GO associations were obtained from EnsemblPlants release 25 (http://archive.plants.ensembl.org/Triticum_aestivum/Info/Index). Only GO terms for Biological Process are shown.

### Hormone profiling analysis

Phytohormones were extracted from individual replicate and quantified by UPLC/ESI-MS/MS at the National Research Council, ACRD-Saskatoon, Canada (https://www.nrc-cnrc.gc.ca/eng/solutions/advisory/plant_hormone.html).

## Results and discussion

### Sumai3 and FL62R1 display higher levels of Type II FHB resistance than the CWRS varieties tested

The *Fusarium graminearum* infection process and disease severity were monitored in the spikelet and rachis. Inoculated spikelets from all four varieties were almost completely bleached or had turned brown by the first week post inoculation (wpi) (S1 Fig). The only difference observed among varieties was in the glume, which were fully bleached in Stettler and Muchmore but remained green in Sumai3 and FL62R1 (S1C and S1D Fig). The browned rachilla observed indicated that *F*. *graminearum* had successfully entered the rachis and was able to infect successive rachises and uninoculated spikelets (S1E Fig).

**Fig 1:**
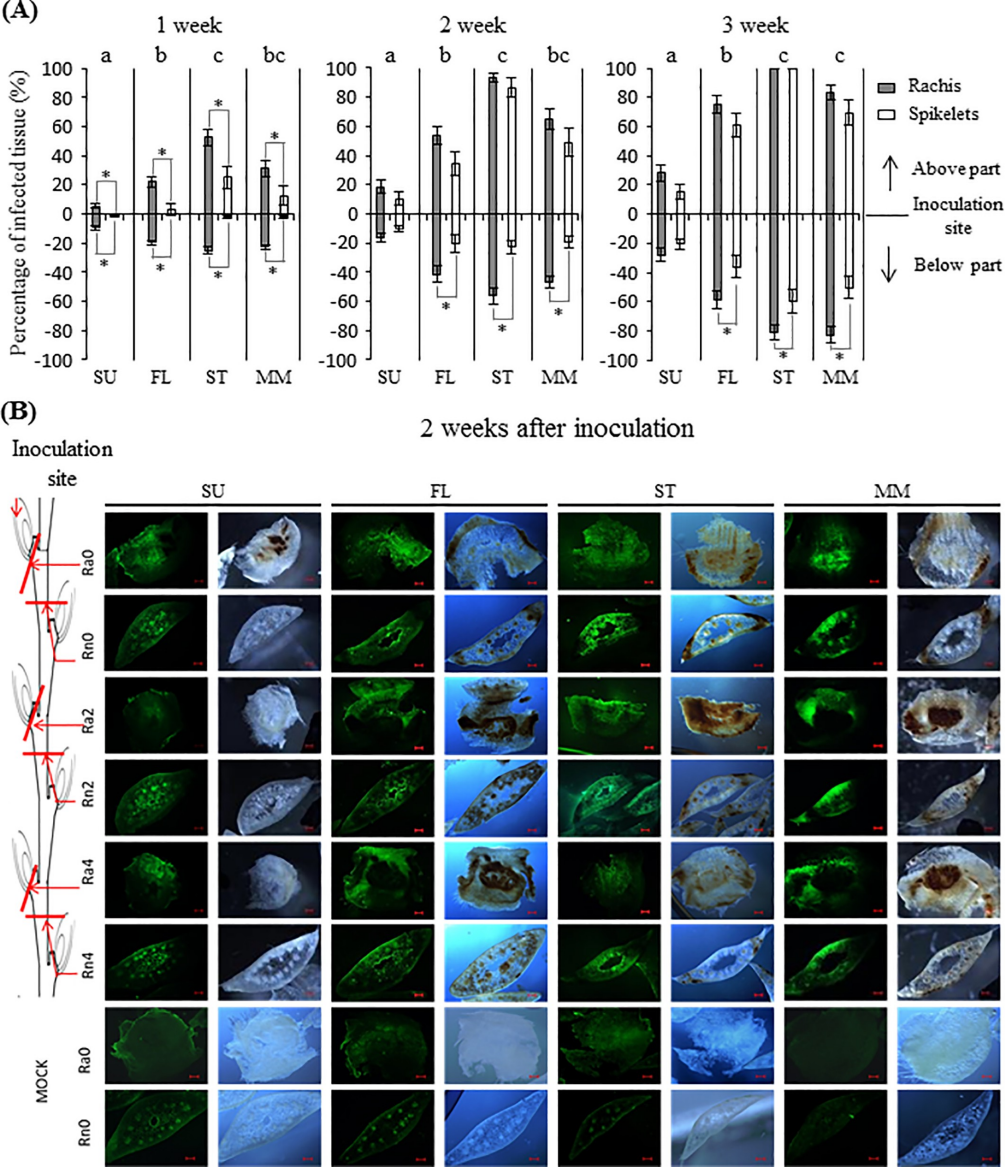
Disease assessment of four wheat varieties. (A) Macroscopic disease symptoms are indicated as the percentage of bleached or brown rachis internodes (grey bar) and spikelets (white bar) above or below the inoculation site over a three-week period. Values represent means ± standard error. For each variety, the Student’s t test was performed between the percentage of infected rachis internodes and spikelets. * indicates P < 0.05. Among the four varieties, a one-way ANOVA of data based on infected rachis internodes was performed at each time point at α = 0.05 to determine significance. Histograms with different letters are statistically different. SU, Sumai3; FL, FL62R1; ST, Stettler; MM, Muchmore. (B) Microscopic observation of the *F*. *graminearum* infection process in the rachis of the four varieties at 2 wpi. The cross-section of rachis internodes and rachillas below the inoculated site were dissected individually and stained with Wheat Germ Agglutinin (WGA). Each sample was photographed separately under fluorescence and light microscope. The scale bar indicates 200 μm. A schematic illustration of a wheat spike is shown on the left-hand side. Red lines indicate the approximate position of hand cross sections in sequential rachises. Each row of photographs represents the same cross section level of each of the four varieties. For the mock treatment, only the adjacent rachilla (Ra0) and rachis internode (Rn0) of mock-treated spikelet were examined and shown in the bottom two rows. Ra, Rachilla; Rn, Rachis internode.

The rachis internodes adjacent to the inoculated spikelets of the three Canadian varieties were already severely bleached or brown at 1 wpi (Fig 1A). Milder symptoms were observed in the corresponding organs of Sumai3 (Fig 1A), suggesting a rachilla-based resistance that prevents or delays hyphae growth into the rachis. By 2 wpi, the percentage of infected or bleached tissue remained low in Sumai3 (Fig 1A); however, dramatic increases in infection symptoms were observed in the other varieties, notably in Stettler, where bleaching above the inoculation site was close to 100% by the end of 3 wpi (Fig 1A). Furthermore, below the inoculation sites, the disease symptoms in the rachis of the three Canadian varieties developed significantly faster and were more severe than in spikelet (Fig 1A). This can be explained by the infection strategy of *F*. *graminearum*, whereby the fungus predominately uses vertical intercellular hyphae to rapidly colonize the vasculature at early stages, and later deploys lateral intracellular hyphae to penetrate the rachilla of uninoculated spikelets [7]. Alternatively, the rachilla may possess resistance mechanisms to prevent the spread of *F*. *graminearum* into uninfected spikelets early in the infection process. At a later stage, *F*. *graminearum* switches to its necrotrophic lifestyle to kill host cells and successfully infect uninoculated spikelet [5].

Overall, the symptoms in Sumai3 were much milder than in the other three varieties at all time points (Fig 1A). The spread of *F*. *graminearum* in FL62R1 was slower than in Stettler at all time points and slower than in Muchmore by 3 wpi (Fig 1A). Thus, among varieties tested, Sumai3 displayed the highest level of Type II FHB resistance, with FL62R1 having moderate resistance, and Stettler and Muchmore being susceptible.

### *F*. *graminearum* growth displayed temporal and organ-specific differences among wheat varieties

To shed light on *F*. *graminearum* proliferation inside the wheat spike, inoculated spikes of the four varieties were dissected and observed microscopically at various time points. Large amounts of hyphae were first detected in the lemma, where *F*. *graminearum* spores started to germinate as early as at 1 dpi. By 2 dpi, massive hyphal growth was observed in the three Canadian varieties (S1A Fig). However, few hyphae were detected in the lemma of Sumai3 until 4 dpi. In the palea, massive hyphae growth was detected at 3 dpi in three Canadian varieties and at 4 dpi in Sumai3 (S1B Fig). In contrast, large amounts of hyphae were not detected in the glumes over the first 4 dpi, except in Muchmore at 4 dpi (S2C Fig). After fully colonizing the spikelet, *F*. *graminearum* hyphae started to infect the adjacent rachilla and spread to the rachis. Extensive hyphal growth in the rachilla was observed as early as 3 dpi in three Canadian varieties, whereas few hyphae were detected in Sumai3 until 6 dpi, suggesting that the rachilla of Sumai3 efficiently prevent *F*. *graminearum* infection (S2D Fig).

Lignification is one of the important cell wall defense responses against *F*. *graminearum* penetration at infection site [28, 29]. Histochemically stained rachilla tissues showed that lignin products were induced after *F*. *graminearum* infection in all four varieties, although at different extents (S2D Fig). For instance, Sumai3 displayed dark red colour at 7dpi, while Muchmore only showed light red colour (S2D Fig).

Following spread to the rachis, cross sections of rachis internodes and rachillas below the inoculated site were examined at 2 wpi (Fig 1B). Consistent with disease severity results, massive *F*. *graminearum* growth was observed to be associated with dark brown lesions as far as the fourth rachis node in the three Canadian varieties (Fig 1B). In the rachis internodes, a large amount of hyphae was detected in sclerenchyma and epidermal cells, resulting in the accumulation of hyphae near the surface and the loss of host cell integrity. In contrast, the dark brown lesions in Sumai3 were detected only in the rachilla adjacent to the inoculation site, but in no other part of the rachis, which remained healthy and retained cell integrity (Fig 1B). Although some hyphae were detected in the vascular bundle in the rachis of Sumai3 internodes, they did not cause browning or lesions, suggesting that biotrophic intercellular hyphae of *F*. *graminearum* persisted in most parts of the rachis of Sumai3 at 2 wpi (Fig 1B).

### Dynamic changes of organ-specific transcriptomes follow inoculation with *F*. *graminearum* as revealed by RNA-seq

To capture the most valuable dynamic transcriptome changes in the four wheat varieties after *F*. *graminearum* infection, a comprehensive RNA-seq experiment was designed according to the above results. Four time points were selected to span key early phases of the *F*. *graminearum*—wheat interaction. These include 1 dpi, when fungal spores had germinated on the inner surface of lemma and palea; 2 dpi, when hyphae started to multiply extensively in the lemma; 3 dpi, corresponding to massive hyphae growth in the lemma and palea and the first detection of hyphae in the rachilla of susceptible varieties; 4 dpi, representing the appearance of macroscopic disease symptoms in the lemma and palea and first browning lesions in the rachilla. Accordingly, the lemma and palea were collected from 1 to 3 dpi (herein referred to as “spikelet”), while the rachilla and rachis adjacent to inoculated spikelet were harvested together from 2 to 4 dpi (“rachis”). Stettler samples at 4 dpi were not collected due to an unexpected problem. In total, 93 samples (31 sets of triplicates) were collected and submitted for RNA sequencing.

On average, 16.6 million reads (range 9.7–24.4 million) per sample were aligned to the wheat and *F*. *graminearum* genomes after quality filtering (S1 Dataset). The percentage of reads mapped to the wheat genome gradually decreased in *F*. *graminearum* inoculated spikelet samples from 1 to 2 dpi, while of the number of reads mapped to the *F*. *graminearum* genome gradually increased (S3 Fig). At 3 dpi, the percentage of reads mapped to wheat dropped dramatically to below 50% in the spikelet, while the proportion of *F*. *graminearum* mapped reads reached up to 63% (S3 Fig). Furthermore, compared to the spikelet, the rachis maintained high host-mapped reads and low pathogen-mapped reads even at 4 dpi, the latest time point analyzed (S3 Fig).

**Fig 2:**
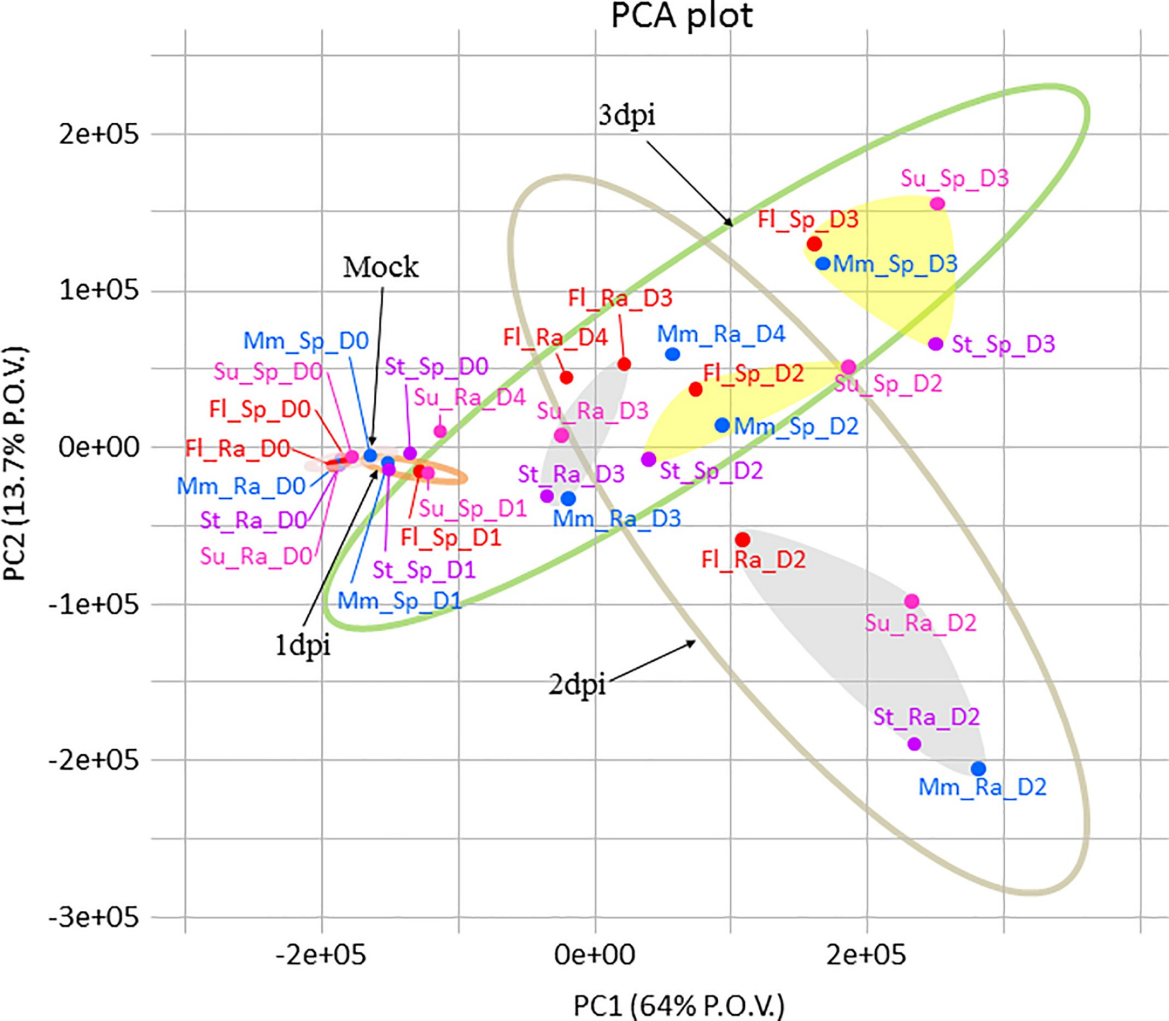
Principal component analysis of the transcriptomes of the two organs from each variety at each time point. Principal component analysis (PCA) was performed using the *prcomp* function from the R base package. Ellipses (confidence level = 0.9) are graphical representations of samples at different dpi. Light yellow areas indicate spikelet-based transcriptomes; grey areas indicate rachis-based transcriptomes. Coloured ovals indicate different time points. Ra, Rachis; Sp, Spikelet; D, dpi.

Principal component analysis (PCA, Fig 2) on differentially expressed genes from RNA-seq datasets revealed a strong separation between the spikelet- and rachis-based transcriptomes at 2 or 3 dpi, indicating distinct transcriptomes following *F*. *graminearum* infection between the two organs.

### Gene ontology enrichment analysis demonstrated that diverse defense mechanisms were expressed faster and more intensely in the spikelet of resistant varieties

**Fig 3:**
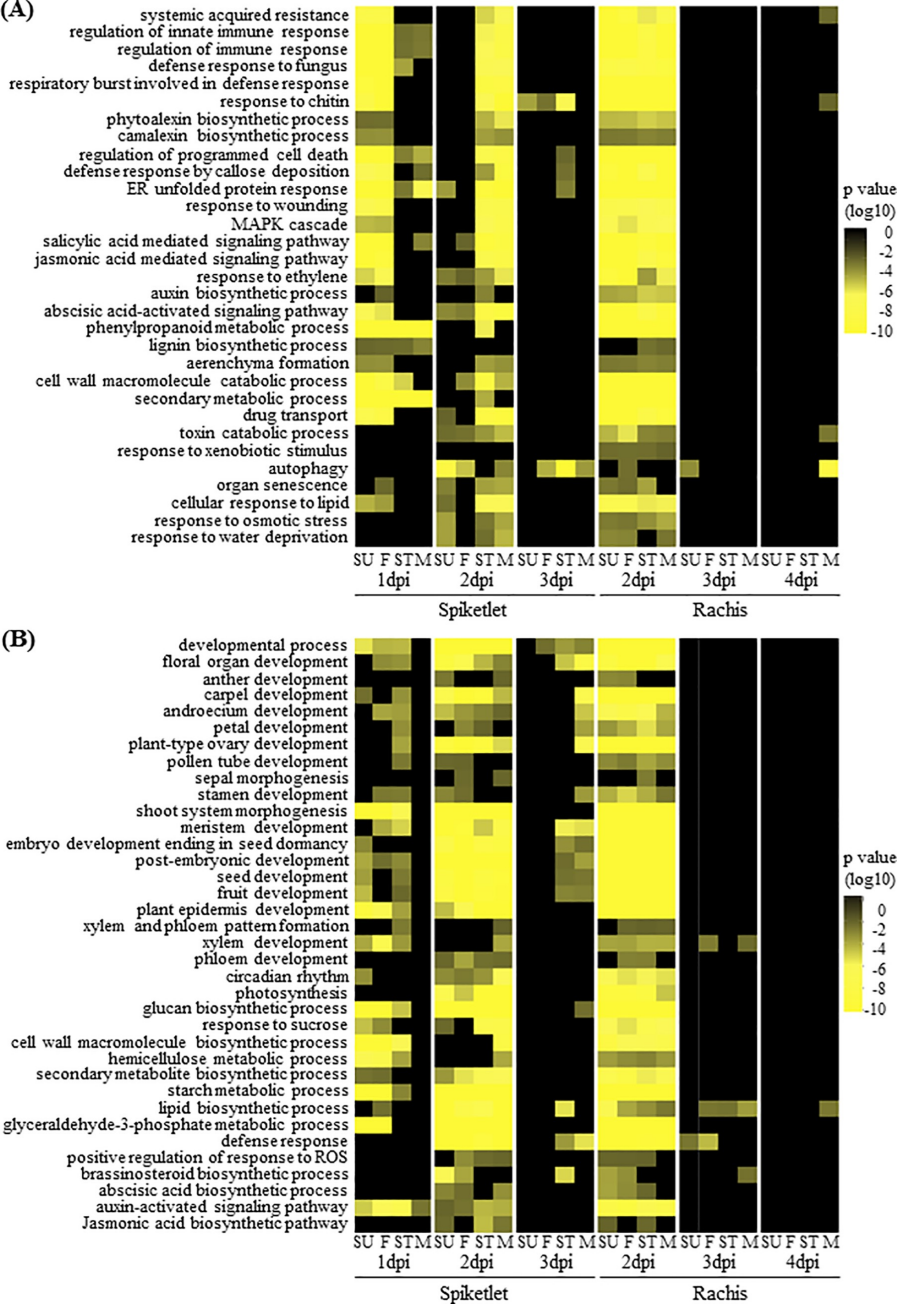
**GO enrichment for DEGs activated (A) and repressed (B) following *F*. *gramineaerum* inoculation.** Heatmap represents the strenghts of the p values of GO term overrepresentation in the *F*. *graminearum*-responsive DEG sets that become significantly changed for the first time at the given time points. Colour index represents level of significance (p values).

A set of differentially expressed genes (DEGs) derived from each variety was identified according to their significance in fold-change expression (p ≤ 0.01) and an additional threshold level cutoff of at least a two-fold change (-1>log2>1) in comparison to the respective mock treatment (S2–S5 Datasets). To investigate the impact of infection timing, DEGs of each organ were further divided into three subgroups according to the time point at which a gene was first differentially expressed after inoculation. According to the expression pattern/trend, DEGs identified at each time point were separated into two classes: up-regulated and down-regulated. Overall, the proportion of down-regulated DEGs at each time point was greater than up-regulated DEGs, especially in Stettler and Muchmore (S4 Fig). Gene Ontology (GO) enrichment analysis on down-regulated DEGs revealed enrichment of the following categories: multiple organ developmental processes, circadian rhythm, photosynthesis, and diverse primary metabolic processes (S6 Dataset and Fig 3B). This suggested that plant host physiology was altered following *F*. *graminearum* infection. Plant hosts may slow down or postpone developmental and photosynthetic processes and reallocate the resources and energy to combat *F*. *graminearum* [15]. Alternatively, *F*. *graminearum* could disrupt host biological processes when invading a host [16].

Consistent with previous studies reviewed in [5, 30], GO enrichment analysis of up-regulated DEGs demonstrated that diverse defense mechanisms were greatly overrepresented during the interaction with *F*. *graminearum* (S6 Dataset and Fig 3A). These defense mechanisms were quickly activated as early as 1 dpi in the spikelet of Sumai3 and FL62R1, but were delayed until 2 dpi in Stettler and Muchmore (Fig 3A). This temporal expression difference is correlated to the resistance levels in the varieties analyzed, implying that the rapid establishment of defense responses can efficiently limit pathogen colonization and defeat pathogens before they launch their virulence tools to attack the plant cell.

Several types of plant hormone-related pathways were significantly affected following challenge with *F*. *graminearum* (Fig 3). While these pathways are not as well understood in wheat as they are in Arabidopsis, on which much of the gene annotation is based, it is noteworthy that Arabidopsis flowers are susceptible to Fusarium infection. The role of hormone signaling pathways in mediating resistance in the Fusarium-Arabidopsis pathosystem has been investigated [31] and changes to the transcriptome in response to Fusarium have been profiled in both wheat and Arabidopsis [30]. Together with functional studies demonstrating conserved features between Arabidopsis and wheat genes conferring resistance to Fusarium (e.g. [32]), available information imply that phytohormone signaling in wheat is conserved features similar to Arabidopsis with regard to defense response against Fusarium pathogen.

To explore the role of hormone pathways for FHB resistance in wheat, genes differentially expressed in each pathway were identified and changes in hormone levels were quantified after *F*. *graminearum* infection.

### Salicylic acid pathways and their role in FHB resistance

**Fig 4:**
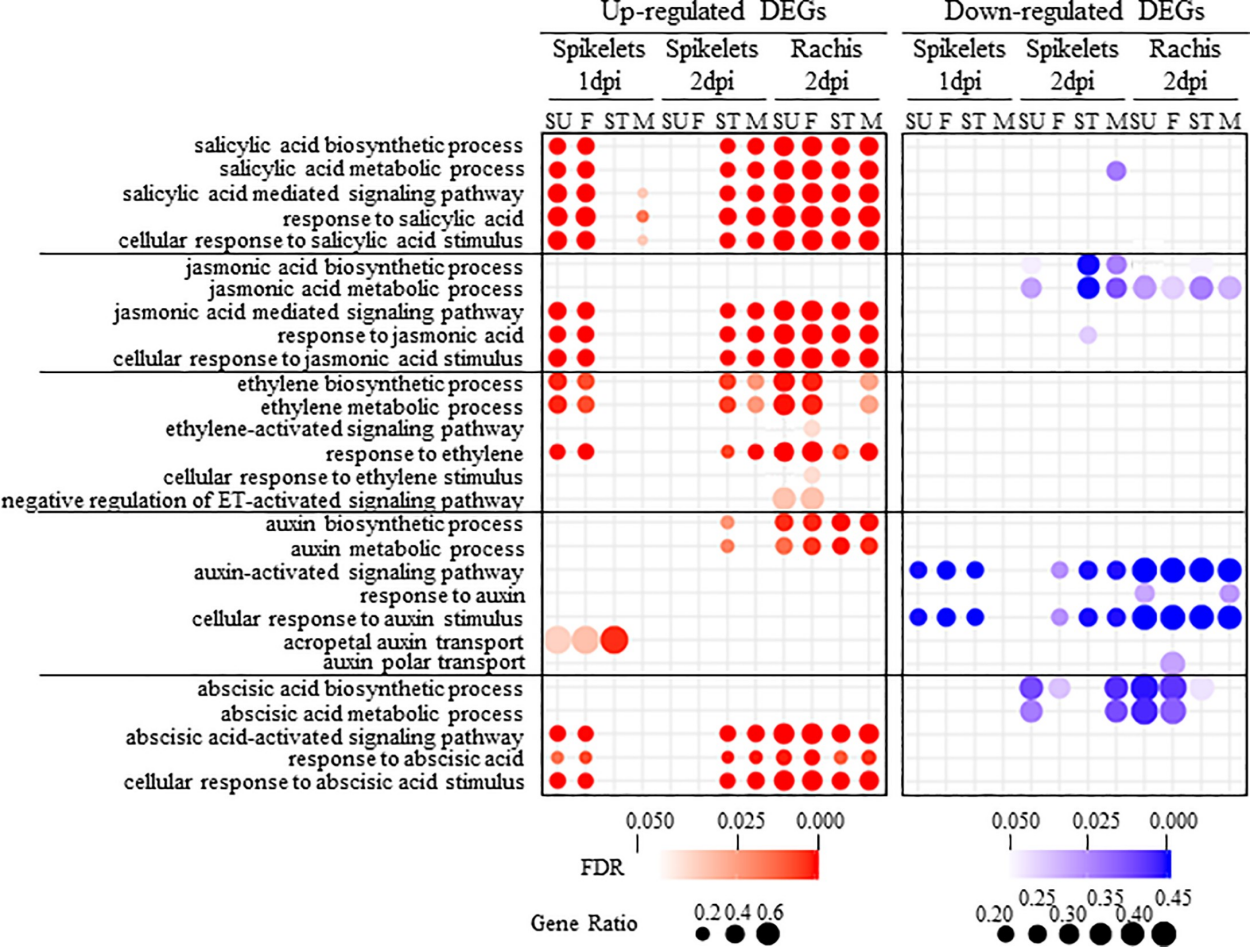
Gene Ontology (GO) enrichment analysis for phytohormone related pathways. Gene ratio equals the number of differentially expressed genes against the number of genes associated with a GO term in wheat genome. The Fisher test was performed to indicate the significance of GO enrichment (FDR < 0.05).

**Fig 5:**
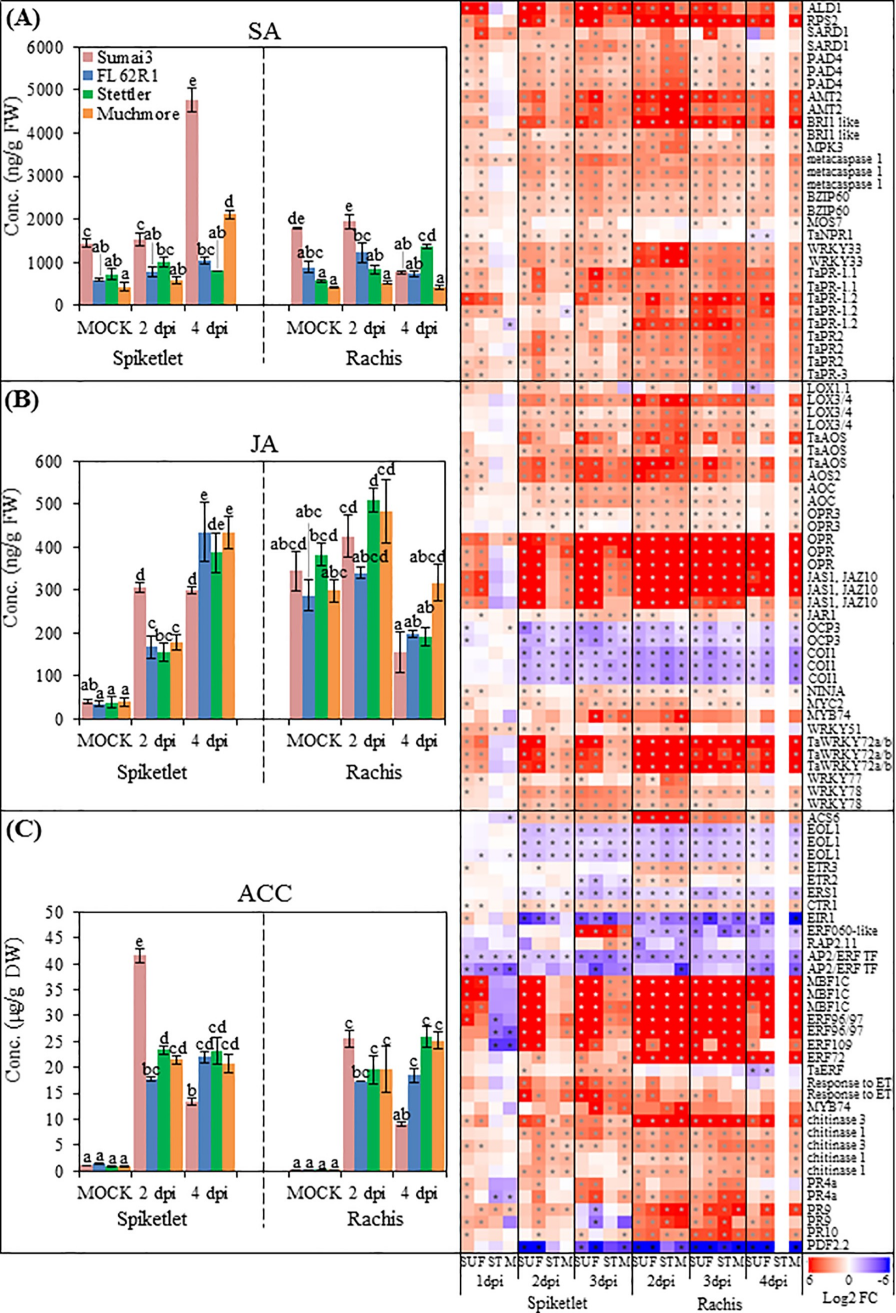
**Hormone and transcriptome profiling of defense related phytohormone pathways: salicylic acid (A), jasmonic acid (B) and ethylene (C) after *F*. *graminearum* infection**. Left panels: hormone content, Values = means ± standard error (n = 3). In each organ, a two-way ANOVA of data was performed at α = 0.05 to determine significance. Histograms with different letters are statistically different. Right panels: extent of differential expression (Log2 fold change). Asterisks (*) denote significant (p ≤ 0.01). A full list of DEGs is provided in S7 Dataset.

Salicylic acid (SA) is a key defense hormone contributing to defense against biotrophic and hemibiotrophic pathogens [33]. Intercellular growth of *F*. *graminearum* at the early infection stage indicates that *F*. *graminearum* has a short biotrophic growth phase before switching to necrotrophy [3, 7]. This suggests the possible implication of the SA pathway in FHB resistance. Indeed, several studies have concluded that the SA pathway plays a critical role in FHB resistance against *F*. *graminearum* [34–36]. Consistent with these reports, GO enrichment analysis of the up-regulated DEGs demonstrated that SA-related pathways and processes were the most overrepresented hormone pathways and among the most significant GO terms overall (Fig 4 and S6 Dataset). For example, in spikelet at 1 dpi, corrected p-values for “cellular response to SA stimulus” and “SA-mediated signaling pathway” reached 10^−12^ in Sumai3 and 10^−11^ in FL62R1. Further investigation found that close to two-thirds (80/123) of SA-related DEGs were up-regulated after *F*. *graminearum* infection in all four varieties (Fig 5A and S7 Dataset). The activation of SA pathway genes in the spikelet of Sumai3 and FL62R1 began quickly, from 1 dpi, whereas corresponding genes in Stettler and Muchmore were not up-regulated until 2 dpi (Figs 4 and 5A).

Some genes displayed differential expression in only one of the organs tested. For instance, the gene annotated to *NPR1* was only up-regulated in the spikelet (Fig 5A). *NPR1* is a key regulator of SA signaling in Arabidopsis [37] and has been shown to contribute to FHB resistance through overexpressing Arabidopsis *NPR1* in wheat [35]. These results suggest that the *NPR1* homologue of wheat may play a role primarily in the spikelet.

To further determine the role of SA in FHB resistance, plant hormone profiling was performed on plant tissues after *F*. *graminearum* infection. The SA level in the spikelet of FL62R1 and Stettler did not change at any of the time points measured, but was dramatically elevated in the Sumai3 spikelet at 4 dpi (Fig 5A). Although SA induction was also observed in Muchmore at 4 dpi, it was lower than in Sumai3. Considering the severe disease symptoms observed in Muchmore (Fig 1), this increase in SA levels does not appear to be sufficient to establish adequate resistance against FHB. This also suggests that additional mechanisms contribute to FHB resistance in addition to SA levels.

Interestingly, both the rachis and spikelet of Sumai3 were found to contain higher basal levels of SA than the other three varieties in mock controls and at the earliest time points sampled following *F*. *graminearum* challenge (Fig 5A). Sorahinobar et al. [36] reported higher basal levels of SA in Sumai3 compared to the susceptible cultivar Falat. Consistently elevated SA concentrations in some plant species have been associated with enhanced disease resistance while resulting in growth inhibition, necrosis and reduced reproductive fitness [38]. Although abnormal development or growth delay were not observed in Sumai3, it will be interesting to determine whether high basal level of SA is associated with its poor agronomics.

### Jasmonic acid pathways and their roles in FHB resistance

Jasmonic acid (JA) is considered to be a critical hormone for plant defense responses against pathogens with a necrotrophic lifestyle [14]. Previous transcriptome analyses revealed that JA signaling pathway genes were highly activated following *F*. *graminearum* infection [34, 39, 40]. Exogenous application of MeJA to wheat heads can enhance resistance against *F*. *graminearum* [35, 39].

To gain better insight into the role of JA towards FHB resistance, GO terms related to JA pathways were analyzed for enrichment among DEGs. Unlike SA biosynthesis GO terms, those related to “JA biosynthesis” and “JA metabolism” were not enriched among up-regulated DEGs, but appeared in down-regulated DEGs from the spikelet of susceptible varieties at 2 dpi following *F*. *graminearum* infection (Fig 4).

The GO terms related to JA downstream pathways, such as “JA mediated signaling pathway” and “response to JA”, were enriched in both organs (Fig 4). Similar to SA signaling genes, the activation of most JA signaling and response pathway genes in the spikelet was faster and stronger in Sumai3 and FL62R1 than in the other varieties (Figs 4 and 5B). Specifically, for genes annotated as *OPR*, *JAS1/JAZ10*, and *WRKY72*, expression levels were at least 3-fold higher in the spikelet of Sumai3 and FL62R1 compared to the other two varieties (Fig 5B and S7 Dataset). Genes encoding negative regulators of JA signaling, including COI1 (CORONATINE INSENSITIVE 1) and OCP3 (OVEREXPRESSOR OF CATIONIC PEROXIDASE 3), were down-regulated in both organs following *F*. *graminearum* infection, with the exception of 1 dpi in the spikelet (Fig 5B). Also similar to results with SA-related genes, relatively little incremental activation of JA response genes in the rachis was observed with time (from 2 to 4 dpi), nor did resistant genotypes display greater differential gene expression in this organ.

Unlike SA, dramatic increases in JA levels were detected in the spikelet of all four varieties after *F*. *graminearum* infection (Fig 5B), despite no overrepresentation of JA biosynthetic genes among the up-regulated DEGs (Fig 4). The uncoupling of RNA-seq and hormone profiling results could be explained by a rapid increase in JA levels at early infection stages which is independent of *de novo* transcriptional activation of biosynthesis genes.

The JA concentration in the Sumai3 spikelet quickly increased and peaked at 2 dpi, which is two-fold higher at that time point than the levels reached in the other three varieties, which peaked at 4 dpi (Fig 5B). This fast response and early induction of JA and its responsive genes in Sumai3 may contribute to the better resistance against *F*. *graminearum* observed in this variety.

### Ethylene pathways and their roles in FHB susceptibility and resistance

The phytohormone ethylene (ET) is dramatically induced in plant tissues after pathogen challenge [41]; however, the role of ET in plant defense is ambiguous due to both positive and negative effects observed during host-pathogen interactions [41]. Apart from its involvement in disease resistance, ET also appears to facilitate disease symptom development by controlling plant chlorosis, senescence, and cell death [42].

The GO enrichment identified three ET-related terms among significantly up-regulated DEGs of the spikelets from Sumai3 and FL62R1 at 1 dpi; “ET biosynthetic processes”, “ET metabolic processes”, and “response to ET” (Fig 4). One day later, these terms appeared as enriched in up-regulated DEGs from the spikelet of Stettler and Muchmore. The significance of GO terms enrichment for “ET biosynthetic processes” and “ET metabolic processes” was greater in up-regulated DEGs of rachises from Sumai3 and FL62R1 than the other varieties at 2 dpi.

Extraction of all ET responsive DEGs indicated that most were up-regulated in the spikelet of Sumai3 and FL62R1 from 1dpi, but not until 3 dpi in Stettler and Muchmore (Fig 5C). Many of these genes, such as pathogenesis-related (PR) genes and chitinase, possess antimicrobial activities and are synergistically regulated by JA [41]. Therefore, it is postulated that induction of these genes contributes to FHB resistance.

To further ascertain the role of ET in response to FHB, we measured the concentration of 1-aminocyclopropane-1-carboxylate (ACC), the immediate precursor of ET, after *F*. *graminearum* infection. To our knowledge, this is the first report to combine RNA-seq profiling with ethylene (ACC) quantification. Levels of ACC were dramatically increased, by at least 12-fold, in both organs of all four varieties after *F*. *graminearum* infection (Fig 5C). In the Sumai3 spikelets, the peak level of ACC at 2 dpi was 40-fold higher than the mock treated samples and was also higher than ACC levels of the other three varieties. Taken together, higher induction of ACC and stronger activation of ET responsive genes in Sumai3 suggested a positive role for ET in FHB resistance during the early stages of infection in this variety.

By 4 dpi, levels of ACC in the spikelet and rachis of Sumai3 were lower than in the other varieties (Fig 5C). It is known that endogenous ET signaling pathways positively regulate organ senescence processes [42]. Bleaching of wheat heads caused by *F*. *graminearum* has long been characterized as premature senescence [1]. The GO term “organ senescence” was highly overrepresented in the up-regulated DEG sets in all four varieties (Fig 3A), indicating that the senescence process was activated in all four varieties after *F*. *graminearum* infection. We presume that elevated levels of ET may contribute to disease development through promotion of senescence during *F*. *graminearum* infection. Consistent with this hypothesis, silencing a key ET signaling gene, *ETHYLENE INSENSITIVE2* (*EIN2*), reduced *F*. *graminearum* -induced FHB symptoms in the moderately susceptible wheat cultivar Bob White [43].

Based on transcript profiling, there have been conflicting conclusions on the role of ET in FHB resistance. Ding et al. [34] and Li and Yen [39] suggested that ET contributes positively to resistance. Consistent with this notion, Ravensdale et al. [44] reported that ET signaling was induced by a resistance-inducing priming treatment. In contrast, Xiao et al. [45] concluded that ET was associated with susceptibility to FHB following an RNA-seq comparison of Wangshuibai and a susceptible mutant. Results from the present study indicate that ET may play a dual role in FHB resistance, which could explain, at least in part, these conflicting reports.

### Auxin pathways and their roles in FHB susceptibility

**Fig 6:**
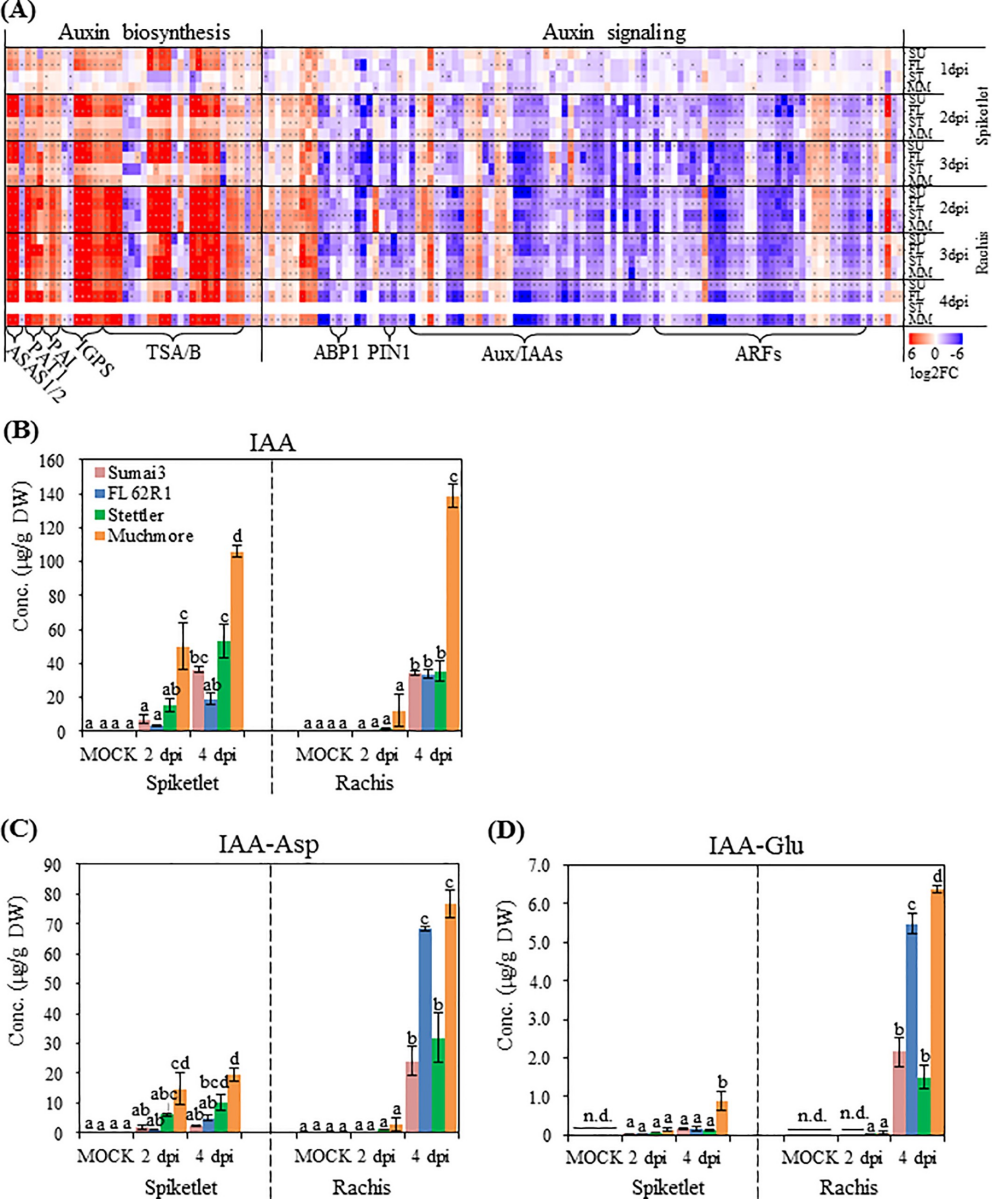
Hormone and transcriptome profiling of auxin (IAA) and two of its amino acid conjugates. (A) extent of differential expression patterns after *F*. *graminearum* infection (Log2 fold change). Asterisks (*) denote significant (p ≤ 0.01). A full list of DEGs is provided in S7 Dataset. (B) IAA, (C) IAA-Asp and (D) IAA-Glu contents in the spikelet and rachis. Values = means ± standard error (n = 3). In each organ, a two-way ANOVA of data was performed at α = 0.05 to determine significance. Histograms with different letters are statistically different.

Elevated levels of auxin in plant tissues have been observed following pathogen infection [46]. In the current analysis, the GO terms “auxin biosynthetic process” and “auxin metabolic process” were highly enriched in the up-regulated DEGs from rachis (Fig 4). Further investigation demonstrated that most auxin biosynthesis genes were up-regulated in both spikelet and rachis after *F*. *graminearum* infection (Fig 6A), indicating that the wheat host auxin biosynthesis pathway was activated following *F*. *graminearum* infection. In the spikelet, stronger expression of these genes started from 1 dpi in Sumai3 and FL62R1 and 2 dpi in Muchmore, whereas weak gene expression was detected in Stettler during the infection period (Fig 6A and S7 Dataset). Compared with the spikelet, the rachis of all four varieties displayed elevated levels of auxin biosynthesis genes at all time points (Fig 6A).

To determine if the activation of auxin biosynthesis genes resulted in the accumulation of auxin, the levels of free indole-3-acetic acid (IAA) were measured after *F*. *graminearum* inoculation. At 2 dpi, substantial IAA accumulation in the spikelet was only observed in Muchmore (Fig 6B). Auxin accumulation in the spikelet of Sumai3 and Stettler occurred at a relatively late time point, 4 dpi. The spikelet of FL62R1 did not display statistically significant changes in free IAA content following *F*. *graminearum* infection. In the rachis, increases in IAA levels at 4 dpi occurred in all four varieties (Fig 6B). Compared to other three varieties, Muchmore accumulated the highest free IAA levels in the rachis. Overall, the transcriptional regulation of auxin biosynthesis genes was not well correlated with the accumulation of free IAA. Specifically, Muchmore accumulated highest levels of free IAA but did not display stronger up-regulation of auxin biosynthesis genes in spikelet. It is noteworthy that *F*. *graminearum* can synthesize auxin [47] and the IAA contents measured by HPLC/MS may include those produced by the fungus.

High IAA content in Muchmore was associated with the greatest susceptibility and the largest amount of *F*. *graminearum* biomass (Fig 1), suggesting that auxin may be associated with disease susceptibility during the interaction with *F*. *graminearum*.

In contrast to auxin biosynthesis pathways, GO terms related to auxin signaling and response to auxin were enriched among down-related DEGs (Fig 4). Enrichment was greater in the rachis of all varieties, regardless of the level of FHB resistance. In the spikelet, enrichment was detected at 1 dpi for Sumai3, FL62R1 and Stettler and at 2 dpi for FL62R1, Stettler and Muchmore. Detailed inspection of the gene lists revealed that they encode both positive and negative regulators of auxin signaling, such as auxin response factors (ARFs) and Aux/IAAs, respectively (Fig 6A). Thus, the outcome of the gene expression patterns on overall auxin signaling was not readily apparent. Interpreting the results was complicated by several factors including: (1) functional specialization between ARFs towards unique aspects of auxin responses; (2) the significant contribution of post-translational regulation, specifically protein degradation, in auxin signaling [48]; and (3) the finding that pathogens can hijack auxin signaling pathways to disrupt plant defense and facilitate pathogen manipulation in host cells [16].

Amino acid conjugated IAA (IAA-AA) has recently been shown to have biological function towards disease susceptibility [49]. We examined the concentrations of IAA-Asp and IAA-Glu in *F*. *graminearum* infected samples. Overall, the rachis accumulated higher levels of these conjugated IAA compared to the spikelet, especially at 4 dpi (Fig 6C and 6D). In the spikelet, with the exception of Muchmore, samples did not accumulate significant levels of IAA-AAs. In the rachis, the accumulation of IAA-AAs occurred at the late time point, 4 dpi. Specifically, Muchmore accumulated a higher concentration of IAA-AAs than the other three varieties. In addition to Muchmore, FL62R1 accumulated a very high level of IAA-AAs at 4 dpi.

These data support a role for auxins in *F*. *graminearum*-induced plant responses, most likely associated with susceptibility. Conjugated IAA accumulated to high levels after *F*. *graminearum* infection in Canadian germplasm, including FL62R1 that possesses intermediate levels of resistance. This illustrates the complexity of auxin metabolism in response to *F*. *graminearum*.

### Abscisic acid pathways and their roles in FHB susceptibility

In addition to its well-documented roles in development and abiotic stress responses, abscisic acid (ABA) has also been implicated in modulated responses to various diseases [50]. Depending on the timing and invasive strategy of the pathogen, ABA can impact resistance either positively or negatively. At the pre-invasive stage, ABA controls stomata closure to prevent pathogen entry [51, 52], whereas at the late post-invasive stage, ABA antagonistically suppresses SA- or JA-dependent defense resistance resulting in susceptibility [53, 54]. Although invasion through stomata openings was occasionally observed with *F*. *graminearum*, the predominant penetration route for the fungus is either via cracked anthers, to grow down the filament into the host plant, or through active penetration of the epidermal cuticle and cell wall of floret [55, 56]. The invasive strategy of *F*. *graminearum* implies that the ABA-mediated stomatal closure defense has little impact on FHB resistance. In contrast, co-application of exogenous ABA with *F*. *graminearum* increases wheat susceptibility [57, 58], suggesting a major negative role for ABA in FHB resistance.

**Fig 7:**
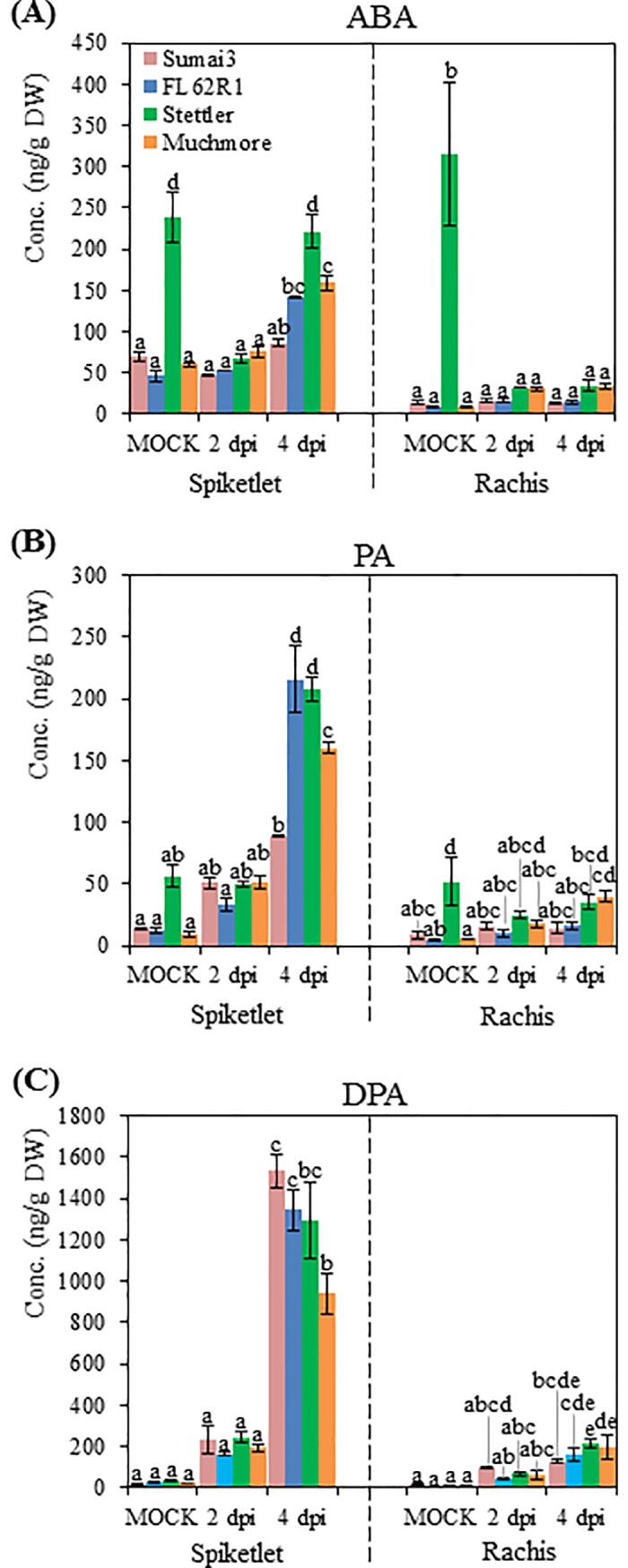
Contents of ABA and its metabolites after *F*. *graminearum* inoculation. ABA (A), PA (B) and DPA (C) contents in the spikelet and rachis. Values = means ± standard error (n = 3). In each organ, a two-way ANOVA of data was performed at α = 0.05 to determine significance. Histograms with different letters are statistically different.

Among the up-regulated DEGs, the GO terms “ABA-activated signaling pathway”, “response to ABA” and “cellular response to ABA stimulus” were significantly overrepresented (Fig 4). The pattern of enrichment was very similar to GO terms related to SA and JA responses and signaling, detected at 1 dpi in the spikelet of resistant varieties, at 2 dpi in the spikelet of susceptible varieties, and at 2 dpi in rachises of all varieties. In contrast, GO terms associated with ABA biosynthetic processes and metabolic processes were enriched in the down-regulated DEGs. Although GO terms for JA biosynthetic and metabolic processes were also enriched in down-regulated DGEs, the patterns were distinct, with GO terms for the ABA processes enriched only in the rachis of FHB resistant varieties and at 2dpi in the spikelet of Sumai3 and Muchmore (Fig 4). More specifically, RNA-seq data indicated that several ABA related genes, including ABA1, ABA2, and ABA3, which have a role in disease resistance [50], were differentially expressed after *F*. *graminearum* infection (S7 Dataset). The ABA concentration increased at 4 dpi in the spikelet of the three Canadian varieties, but not in the resistant cultivar Sumai3 (Fig 7A). ABA levels did not accumulate in the rachis of the four varieties after *F*. *graminearum* inoculation. Interestingly, Stettler demonstrated high basal levels of ABA in both spikelet and rachis without *F*. *graminearum* inoculation (Fig 7A).

ABA catabolic pathways are conventionally thought of as routes for ABA inactivation. Nevertheless, increasing reports imply that some ABA metabolites, such as phaseic acid (PA), possess ABA-like biological activity ranging from the regulation of stomata closure to inhibition of seed germination [59, 60]. Eventually, PA is metabolically converted to dihydrophaseic acid (DPA), which is inactive in various bioassays [61].

Similar to the ABA pattern, PA and DPA accumulated to high levels at 4 dpi in the spikelet of the four varieties (Fig 7B and 7C). At this time point, PA levels in Sumai3 was significantly lower than in other varieties (Fig 7B). In contrast, Sumai3 accumulated the highest DPA level among varieties at 4 dpi (Fig 7C). Greater conversion of the negative regulator PA into the inactive DPA in Sumai3 may contribute to greater resistance to FHB.

## Conclusions

Building on the existing well-accepted point inoculation assay, a more comprehensive and precise disease evaluation strategy for FHB was developed. To accurately capture disease development on wheat spikes, extra disease symptom parameters were recorded, including the number of brown or bleached rachis internodes, the assessment of disease symptoms above or below the inoculation site, and the combined macroscopic and microscopic observations. This analysis demonstrated that distinct organs in the spike differentially responded to *F*. *graminearum* infection, which allowed organ-specific analyses to be performed.

Overall, transcript profiling results obtained were consistent with previous reports reviewed in [30] that wheat responds to *F*. *graminearum* infection in much the same way as observed for other plant-pathogen systems, including faster and more pronounced activation of well-characterized defense responses in resistant germplasm. However, the availability of a reference wheat genome, the use of RNA-seq technology, and organ-specific profiling yielded more precise and meaningful information than many previous studies. Combined with the comprehensive quantification of multiple plant hormones, detailed information on the potential role of these growth regulators could be inferred, including:

1. SA and JA play predominantly positive roles in FHB resistance, whereas auxin and ABA may be associated with susceptibility to FHB. ET appears to play a dual role during the interaction with *F*. *graminearum*. Although previous transcriptome analyses emphasized a positive role of JA for FHB resistance [34, 39, 40], no other genome-wide transcript profiling studies has highlighted a major role of SA.
2. A number of DEGs were commonly involved in more than one hormone pathway (S7 Dataset and S5 Fig), indicating possible crosstalk between these pathways in response to challenge with Fusarium. SA and JA shared largest number of common genes the DEG sets while three DEGs annotated as ABA3 were detected in all five studied hormone pathways. Functional validation will be required to determine the impact of these genes on disease resistance before targets for crop improvement can be identified.
3. Patterns of hormone-related gene expression and levels of hormones differ substantially between the spikelet and the rachis, with the spikelet displaying faster and greater activation of most hormone-related signaling and response genes in resistant varieties, which was not observed in the rachis. Limited differences in hormone-related gene expression were detected in the rachis at different times after *F*. *graminearum* challenge or between varieties, and no increase in SA or JA levels were detected in the rachis following *F*. *graminearum* infection.
4. Overall patterns of pathway regulation following *F*. *graminearum* challenge differed considerably between phytohormones with three patterns emerging: (i) up-regulation of both biosynthetic/metabolic and signaling/response category pathways, observed for SA and ET; (ii) down-regulation of biosynthetic/metabolic pathways and up-regulation of signaling/response pathways, seen with JA and ABA; (iii) up-regulation of biosynthetic/metabolic pathways and down-regulation of signaling/response pathways, witnessed for auxin. Comparisons with other genome-wide gene expression datasets will be needed to determine the generality of these patterns, while additional analysis will be required to assess their significance. This could include pharmacological tests involving the application of plant hormones or antagonists, and genetic studies to manipulate the expression and function of key genes within target pathways.
5. Overwhelmingly, the same DEGs were identified in all four varieties as well as both organs studied; however, dramatic differences were detected in the levels of expression among varieties and between organs. These are likely the key determinants between resistance and susceptibility.

## Supporting information

S1 Fig The macroscopic symptoms of *F. graminearum* infection on spikelets at one week after point inoculation. Photographs were taken of the entire inoculated spikelet and adjoining rachis (A), and of the individually excised organs of the inoculated spikelet, lemma (B), glume (outside, C; inside, D), and rachilla (E).
(TIF)

S2 Fig Microscopic observation of the *F. graminearum* infection process in the inoculated spikelets. The individually excised organs of inoculated spikelet, lemma (A), palea (B), glume (C), and rachilla (D) were examined at different days after inoculation. All organs were stained with WGA; rachillas were additionally stained with the phloroglucinol-HCl solution to indicate lignification. Samples were separately photographed under fluorescence (left-side photo) and light (right-side photo) microscopy. Fungal hyphae appear in green under fluorescence. Lignin appears as red under light microscopy. The scale bar indicates 200 μM. Each row of photographs represents the same time point among four varieties.
(TIF)

S3 Fig Overview of percentage of RNA-seq reads mapped to wheat and *F. graminearum* genomes. (TIF)

S4 Fig Time course-based summary of DEGs sets, using the first time the gene becomes significantly activated (up) or repressed (down). Red bars represent up-regulated DEGs; green bars represent down-regulated DEGs. The Y axis represents the number of DEGs; X axis represents time points.
(TIF)

S5 Fig Comparison of DEGs annotated with GO terms for defense-related hormone pathways. Venn diagrams were made using http://bioinformatics.psb.ugent.be/webtools/Venn/. Pathways related to phytohormones salicylic acid (SA), jasmonic acid (JA), ethylene (ET), abscicic acid (ABA), and auxins were considerred.
(TIF)

S1 Dataset Summary of RNA-seq read mapping to wheat and *F. graminearum*. (XLSX)

S2 Dataset List of DEGs in Sumai3. (XLSX)

S3 Dataset List of DEGs in FL62R1. (XLSX)

S4 Dataset List of DEGs in Stettler. (XLSX)

S5 Dataset List of DEGs in Muchmore. (XLSX)

S6 Dataset List of GO terms for time course based DEGs sets. (XLSX)

S7 Dataset List of DEGs in defense related pathways. (XLSX)
