# Supplementary figures and images for: Integrated transcriptome and hormone profiling highlight the role of multiple phytohormone pathways in wheat resistance against fusarium head blight
Source: PLoS One. 2018 Nov 7;13(11):e0207036. doi: 10.1371/journal.pone.0207036 (PMC6221353; doi:10.1371/journal.pone.0207036)

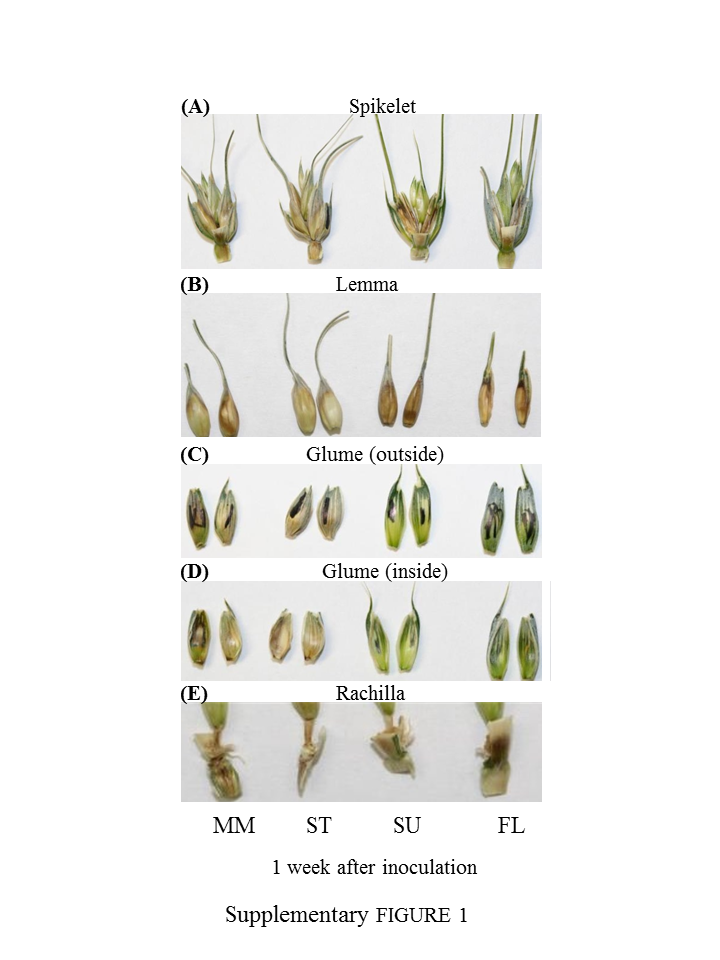

Supplement: S1 Fig — Photographs were taken of the entire inoculated spikelet and adjoining rachis (A), and of the individually excised organs of the inoculated spikelet, lemma (B), glume (outside, C; inside, D), and rachilla (E). (TIF) [file pone.0207036.s001.TIF]

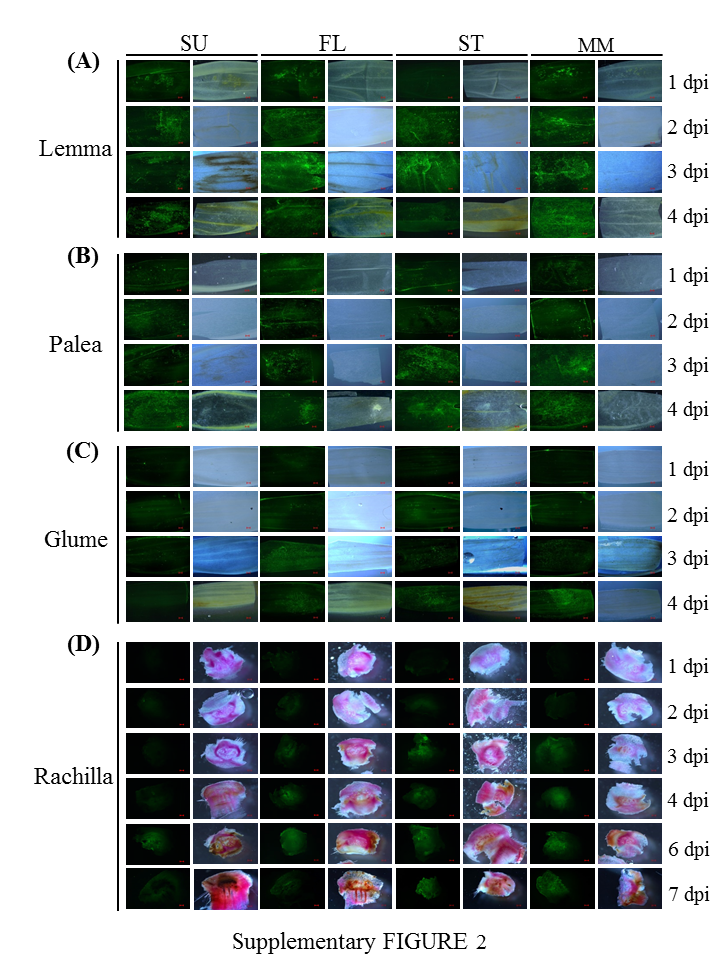

Supplement: S2 Fig — The individually excised organs of inoculated spikelet, lemma (A), palea (B), glume (C), and rachilla (D) were examined at different days after inoculation. All organs were stained with WGA; rachillas were additionally stained with the phloroglucinol-HCl solution to indicate lignification. Samples were separately photographed under fluorescence (left-side photo) and light (right-side photo) microscopy. Fungal hyphae appear in green under fluorescence. Lignin appears as red under light microscopy. The scale bar indicates 200 μM. Each row of photographs represents the same time point among four varieties. (TIF) [file pone.0207036.s002.TIF]

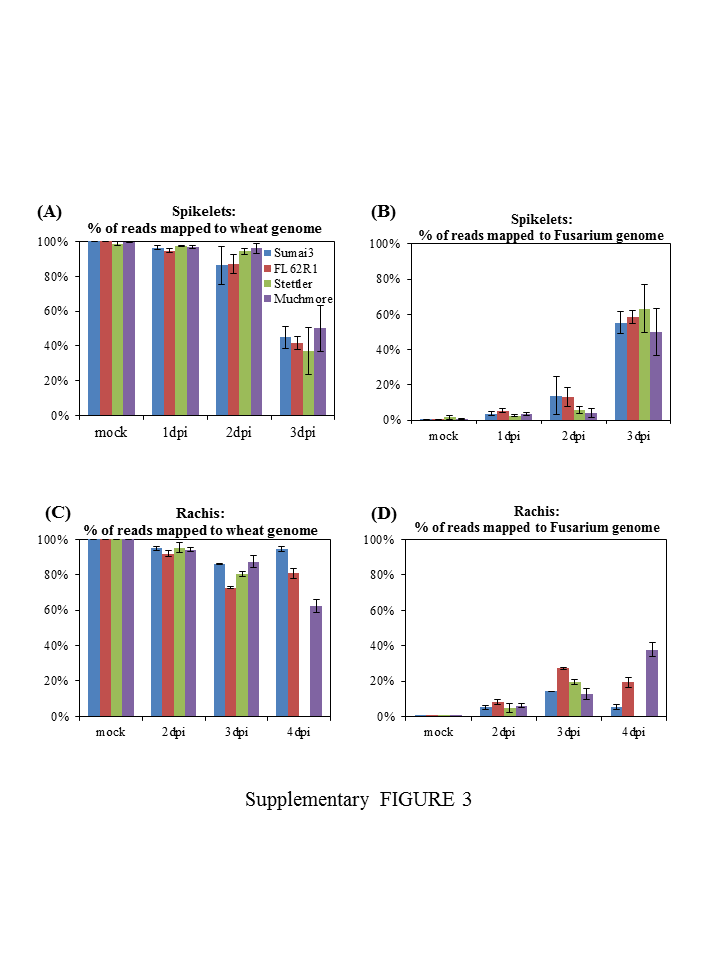

Supplement: S3 Fig — (TIF) [file pone.0207036.s003.TIF]

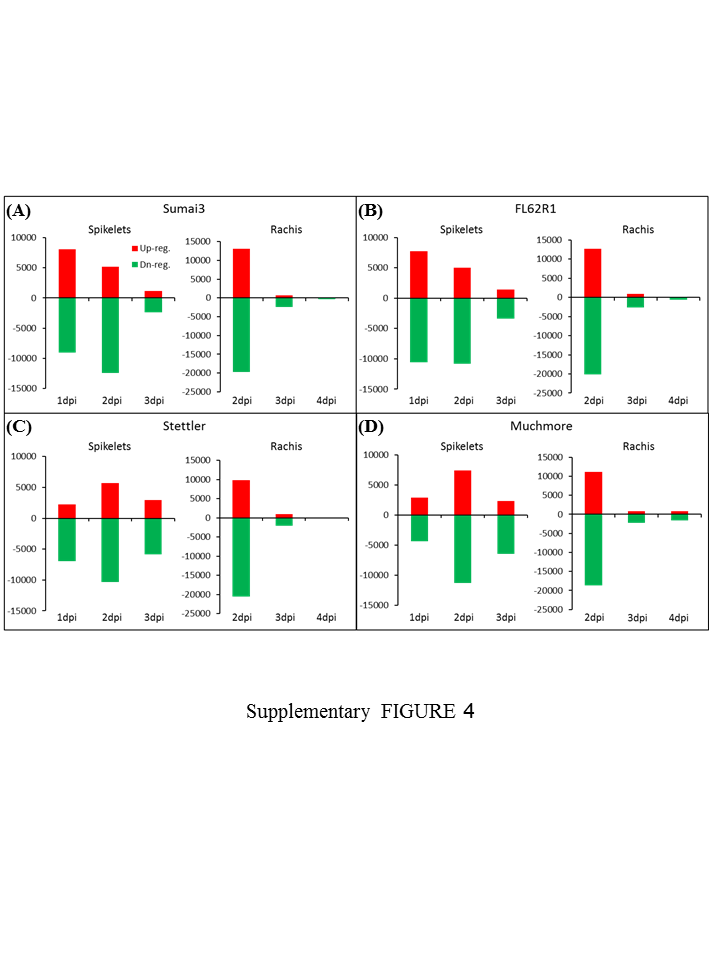

Supplement: S4 Fig — Red bars represent up-regulated DEGs; green bars represent down-regulated DEGs. The Y axis represents the number of DEGs; X axis represents time points. (TIF) [file pone.0207036.s004.TIF]

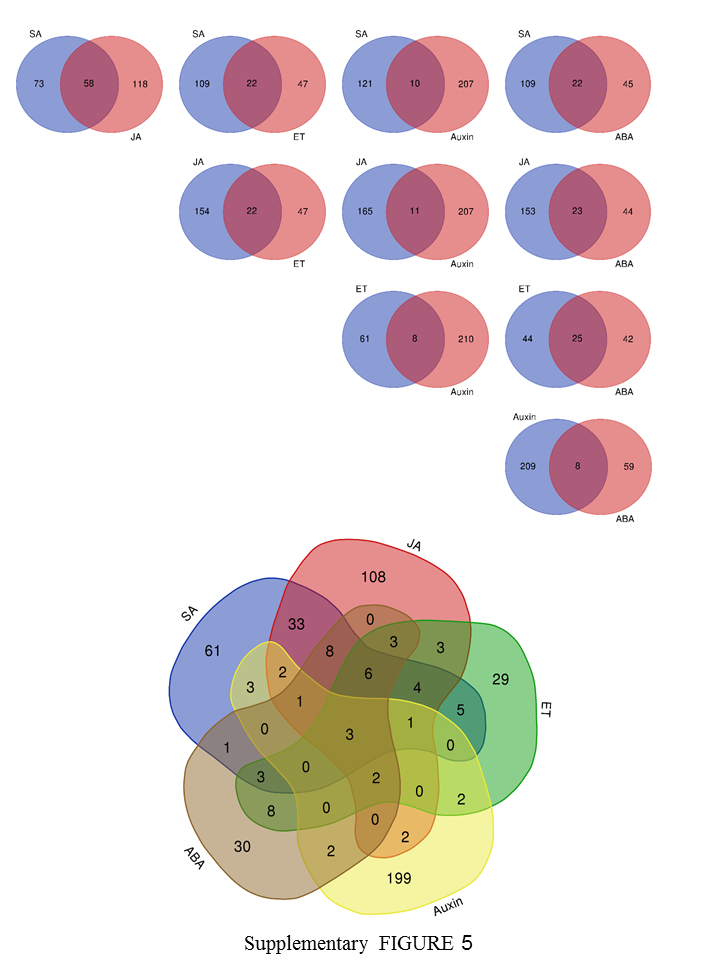

Supplement: S5 Fig — Venn diagrams were made using http://bioinformatics.psb.ugent.be/webtools/Venn/. Pathways related to phytohormones salicylic acid (SA), jasmonic acid (JA), ethylene (ET), abscicic acid (ABA), and auxins were considerred. (TIF) [file pone.0207036.s005.tif]
